# Sensor-based fall risk assessment in older adults with or without cognitive impairment: a systematic review

**DOI:** 10.1186/s11556-021-00266-w

**Published:** 2021-07-09

**Authors:** Jelena Bezold, Janina Krell-Roesch, Tobias Eckert, Darko Jekauc, Alexander Woll

**Affiliations:** 1 Institute of Sports and Sports Science, Karlsruhe Institute of Technology, Engler-Bunte-Ring 15, 76131 Karlsruhe, Germany; 2 Department of Health Sciences Research, Mayo Clinic, Rochester, MN USA

**Keywords:** Risk of falling, Wearable sensors, Elderly, Cognition, Dementia

## Abstract

**Background:**

Higher age and cognitive impairment are associated with a higher risk of falling. Wearable sensor technology may be useful in objectively assessing motor fall risk factors to improve physical exercise interventions for fall prevention. This systematic review aims at providing an updated overview of the current research on wearable sensors for fall risk assessment in older adults with or without cognitive impairment. Therefore, we addressed two specific research questions: 1) Can wearable sensors provide accurate data on motor performance that may be used to assess risk of falling, e.g., by distinguishing between faller and non-faller in a sample of older adults with or without cognitive impairment?; and 2) Which practical recommendations can be given for the application of sensor-based fall risk assessment in individuals with CI? A systematic literature search (July 2019, update July 2020) was conducted using PubMed, Scopus and Web of Science databases. Community-based studies or studies conducted in a geriatric setting that examine fall risk factors in older adults (aged ≥60 years) with or without cognitive impairment were included. Predefined inclusion criteria yielded 16 cross-sectional, 10 prospective and 2 studies with a mixed design.

**Results:**

Overall, sensor-based data was mainly collected during walking tests in a lab setting. The main sensor location was the lower back to provide wearing comfort and avoid disturbance of participants. The most accurate fall risk classification model included data from sit-to-walk and walk-to-sit transitions collected over three days of daily life (mean accuracy = 88.0%). Nine out of 28 included studies revealed information about sensor use in older adults with possible cognitive impairment, but classification models performed slightly worse than those for older adults without cognitive impairment (mean accuracy = 79.0%).

**Conclusion:**

Fall risk assessment using wearable sensors is feasible in older adults regardless of their cognitive status. Accuracy may vary depending on sensor location, sensor attachment and type of assessment chosen for the recording of sensor data. More research on the use of sensors for objective fall risk assessment in older adults is needed, particularly in older adults with cognitive impairment.

**Trial registration:**

This systematic review is registered in PROSPERO (CRD42020171118).

**Supplementary Information:**

The online version contains supplementary material available at 10.1186/s11556-021-00266-w.

## Introduction

With increasing age, cognitive function and motor abilities decline and risk of falling increases [1]. One in three individuals over the age of 65 years experiences one or more falls in any given year, and this prevalence increases to 40% among individuals aged 80 years and older [2]. Falling often leads to severe injuries, hospitalization, loss of autonomy in activities of daily living, reduced quality of life, and an accelerated need for help in older adults [2, 3]. Furthermore, fall-related mortality increases with age [4]. Individuals with cognitive impairment (CI) fall twice as often as their unimpaired peers and have a threefold increased risk of suffering a bone fracture after a fall [5, 6].

A number of motor disabilities are known to be related to a higher risk of falling [7, 8], for example negative changes in gait under single and dual task conditions, balance or lower extremity strength [1, 3, 9]. In individuals with CI, accelerated decline of motor performance is associated with an increased risk of falling as compared to cognitively unimpaired older adults [10, 11].

Physical exercise interventions in fall prevention are promising, as they are associated with improved gait performance, balance and mobility in older adults [12, 13]. Therefore, the identification and quantification of modifiable fall risk factors may be important for the design of effective physical rehabilitation or fall prevention programs that specifically address the needs and burdens of older individuals at high risk of falling [14]. Since falling events in geriatric settings are usually recorded by fall diaries implying a higher risk to recall bias [15], there is a need for the identification and investigation of fall-related factors that may serve as more reliable indicators of a person’s fall risk than recorded total number of prior falls.

To date, such key factors of motor performance are commonly assessed using questionnaires, scales or objective clinician-rated functional performance tests, such as the Short Physical Performance Battery (SPPB) [16] or the Timed- Up and Go Test (TUG) [17], usually evaluated by timekeeping or scoring. Nevertheless, not all of these assessments are feasible, particularly for older individuals with CI [18] and the scales often show a high inter-rater variability [19].

Within the last ten years, wearable technology providing objective data has become more prevalent in clinical settings [20]. Small and lightweight body-worn sensors like accelerometers or gyroscopes hold great promise in the field of fall detection, but also in fall risk assessment [2, 21]. Moreover, these devices are more economic than gold standard methods of motion analysis systems [22] and more applicable in clinical and non-clinical settings as their high level of portability allows the examination of human motion in field instead of laboratory testing [23].

Fall detection using wearable sensors can reduce fall-related injuries and healthcare costs, and is often used as an alarm system in case of an emergency, i.e. accidental fall. The recognition of fall events can be used to trigger helping systems (e.g. alarming signals to caregivers) and may help to understand the mechanism underlying the fall incident [24, 25]. Thereby, fall detection systems may prevent an individual from remaining in a helpless position on the floor for an extended period of time [25]. A recent review on single and multiple sensor-based fall detection concluded wearable sensor-based solutions to be of accuracy to detect fall-events in older adults [25]. Nevertheless, fall detection systems using multiple input sources may lead to high costs and their use is often restricted to indoor locations [25, 26]. Furthermore, fall detection systems may help to identify external fall risk (e.g., uneven ground) but they are limited in providing information about internal fall risk factors, e.g., dysfunctional patterns of gait or required motor tasks that are of interest to conceptualize fall prevention strategies. To this end, using wearable sensors for fall risk assessment may comprehensively capture characteristics of different motor tasks allowing an estimation of human motion (e.g. spatio-temporal characteristics of balance or gait or transfer performance from sitting to standing) [20, 27].

Current reviews on body-worn sensors for the assessment of fall risk focus either on methodological aspects such as applied classification methods and model assessment outcomes, or on practical aspects such as type, number and location of sensors and are often limited to older people without CI [2, 20, 27–29]. Moreover, most of published reviews are limited to either a supervised or a unsupervised setting or included studies with other quantitative measures like instrumented walkways or motion capturing systems [30].

Therefore, the overarching aim of the present systematic review was to provide an overview and update of the existing body of literature that examined the feasibility of body-worn sensors for the assessment of motor fall risk among older adults. Furthermore, we deliberately aimed at including studies that focused on older adults with CI to give practical advice on the use of wearable sensors in individuals with CI. To this end, we addressed two specific research questions: 1) Can wearable sensors provide accurate data on motor performance that may be used to assess risk of falling, e.g., by distinguishing between faller and non-faller in a sample of older adults with or without cognitive impairment?; and 2) Which practical recommendations can be given for the application of sensor-based fall risk assessment in individuals with CI?

The following paragraphs contain a detailed description of the methodological procedure of this systematic review, i.e. search strategy, study selection and data synthesis. In the results section we present study design, detection of fall status, use of sensors to assess fall risk, and classification models of the included studies. Furthermore, results of studies including individuals with CI are presented separately. Finally, we summarize our findings in accordance with the objectives with this systematic review and discuss the strengths and limitations as well as practical implications.

## Methods

### Protocol

We followed the Preferred Reporting Item for Systematic review and Meta-Analysis (PRISMA) guidelines in preparing this systematic review [31]. Furthermore, we registered this review in PROSPERO (CRD42020171118).

### Search strategy and eligibility criteria

We performed a literature search using PubMed, Web of Science and Scopus databases with no time filter set. Articles were searched using the following combination of key words: (fall risk OR fall risk factor*) AND (sensor* OR objectively measured OR objective measurement OR acceleromet*). Population or cognitive status were not included in the search term because we did not want to restrict our results, for example by potentially excluding articles that had mixed study populations. Rather, we deliberately kept our literature search as inclusive as possible. No filter was applied at this stage. The complete literature search can be found in supplementary material (Additional file 1). We screened the reference lists of included articles for relevant secondary literature. The initial database search was conducted in July 2019 with an updated search in August 2020. The following inclusion criteria for the studies were defined:

- Original research articles in peer reviewed journals in English language;
- Studies including individuals with a mean age of 60 years or older, with or without presence of CI;
- Studies assessing fall-related motor performance using body-worn, sensor-based tools in a clinical or community-based setting or in nursing homes, and;
- Studies sub-dividing their sample into fallers and non-fallers, or into individuals at high and low fall risk based on prospective or retrospective falls, clinical assessments or the combination of these methods.

Studies were excluded if a) the mean age of the reported sample was younger than 60 years, b) the individuals showed concomitant severe chronical conditions (e. g., stroke, Parkinson’s Disease), and c) only environmental sensor-based systems (e.g. 2D video analysis) were applied. As the focus on fall risk assessment may provide more pertinent information that enables the design of new preventive approaches, i.e., physical exercise interventions, we also excluded studies with the purpose of fall detection.

### Study selection

After detection and removal of duplicates, two authors (JB and JKR) independently screened all titles and abstracts of the literature search. Both authors repeated this process by screening the abstracts (or full texts if more information was needed) of the remaining articles based on the above defined inclusion criteria. In case of any discrepancy, a third author (TE) was consulted. If there was disagreement about the final inclusion of an article, the third author read the full text and made a final decision. Literature management was performed using Citavi Software (Version 6.3.0.0, Swiss Academic Software GmbH).

### Data extraction and data synthesis

First, relevant data of the included studies were independently extracted and systematically recorded by two authors (JB and JKR) using a standardized data extraction form. Second, the collected data was cross-checked to ensure complete and correct data extraction. We extracted first author’s name, publication date, study design, sample size and population characteristics (i.e., sex, age, cognitive status). We also collected information on fall classification methods that was used to differentiate between fallers and non-fallers or individuals at high and low fall risk. Additionally, the following specific characteristics about the use of body-worn sensors were collected: type of sensor(s), location of sensor(s), activities while sensor data were collected (e.g., during clinical assessment of the TUG) and the parameters of sensor data collected. Furthermore, results of prediction models were extracted and accuracy, sensitivity and specificity were extracted. Accuracy is defined as the ability to discriminate between fallers and non-fallers or between people at high and people at low fall risk. Sensitivity describes the true-positive proportion and specificity describes the true-negative proportion. An accuracy of 50% means that no discrimination exists and that this performance can be achieved by chance [32]. After data extraction, one author (JB*)* synthesized the data.

### Assessment of methodological quality

Two authors (JB and JKR) independently assessed the methodological quality of each study included in this systematic review using the Newcastle-Ottawa Scale (NOS) for cross-sectional and for prospective or cohort studies [33, 34]. The scale uses an evaluation system with stars across three categories, i.e. selection (cross-sectional: 0–5 stars; prospective: 0–4 stars), comparability (cross-sectional: 0–2 stars; prospective: 0–2 stars) and outcome (cross-sectional: 0–3 stars; prospective: 0–3 stars). A higher number of total stars (cross-sectional: range 0–10; prospective: 0–9) reflects a higher study quality with regard to the respective categories.

## Results

**Fig. 1:**
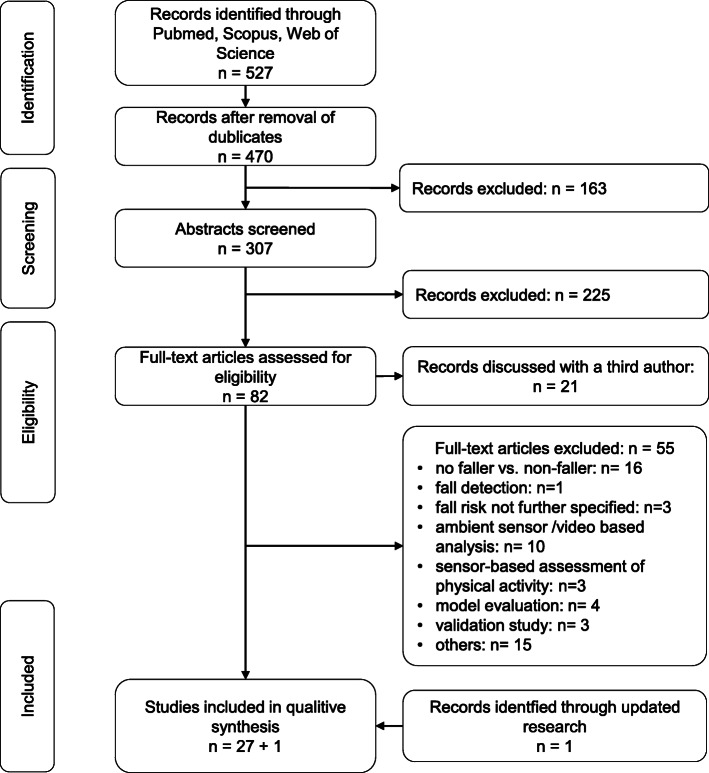
Flow chart of the literature search

After the identification of 527 studies and the screening of 307 abstracts, 82 full-text articles were checked for the inclusion criteria. Finally, a total of 27 studies were included in this systematic review (Fig. 1). An updated search in July 2020 resulted in one additional article. Screening the reference lists resulted in no additional articles.

### Study design and sample characteristics

The included studies were published between 2009 and 2020. Sixteen of the included studies had a cross-sectional design, ten studies had a prospective / longitudinal design and two studies combined cross-sectional with prospective design. The follow-up period of included prospective studies differed between 2 and 24 months. Seventeen studies were conducted in a supervised setting (e. g., clinical setting), whereas six studies collected unsupervised sensor data during daily life. Five studies combined the two settings.

A total of 2896 participants (range = 35–303; 65% females) were included in the studies. Twenty studies included community-dwelling participants, eight studies were conducted in patients who were hospitalized or residing in a geriatric care facility. The mean age of the older participant groups ranged between 68 and 86 years. Two studies [19, 35] included younger control groups with a mean age between 21 and 35 years, however, we did not consider these groups for the purpose of this review. CI was an exclusion criterion in most of the studies (*n* = 14) and only one study deliberately focused on older people with dementia [36]. To determine cognitive status, the Mini-Mental-State Examination (score 0–30) [37], the MiniCog (score 0–5) [38] or the Short Orientation-Memory-Concentration Test (score 0–26) [39] were used. The remaining studies did not explicitly state CI as an exclusion criterion but required to be able to understand the test instructions. Further information about study characteristics and the main findings of the studies is presented in Table 1.

**Table 1:** Study design, sample characteristics and main results

Author, year	Study design, sample including number of participants, mean age (SD) and sex	Cognitive Status	Record of falls/ fall history	Main findings
Bautmans, 2011 [40]	Cross-sectional Community-based Total *n* = 121, 80 (5), 50% female; Younger adults *n* = 40, 22(1), 50% female	Cognitively intact according to MMSE (MMSE≥24)	Retrospective 6 months, Tinetti Assessment Tool, Timed-Up and Go HFR n = 40, LFR n = 41	- Participants with HFR showed slower gait speed (*p* < 0.05) - With cut-off value 1.58 m/s gait speed discriminates between HFR and LFR with 78% sensitivity and 76% specificity
Bizovska, 2018 [43]	Prospective study Community-based Total *n* = 131, 71 (6), 82% female	CI as exclusion criterion	Prospective 1 year SF = 35, MF = 15, NF = 81	- Trunk medial-lateral acceleration in short-term Lyapunov exponent differed between MF and NF (*p* < 0.05) but not after Bonferroni correction; - Poor MF predictive ability of trunk medio-lateral short-term Lyapunov exponent but results improved when combining with clinical examination
Brodie, 2017 [59]	Cross-sectional Community-based Total *n* = 96, 75 (8), 59% female	CI as exclusion criterion according to MiniCog	Retrospective 12 months F = 33, NF = 63	- Fallers showed significantly reduced gait endurance and increased within-walk variability (p < 0.05)
Brodie, 2015 [47]	Cross-sectional Community-based Total n = 96, 80 (4), 67% female	No information about CI	Retrospective 1 year, Physiological Profile Assessment Tool F = 35, NF = 61	- 8-step mediolateral harmonic ratio identified significant differences in between F and NF based on age, walking speed and physiology (*p* < 0.05)
Buckinx, 2015 [48]	Prospective study Nursing homes Total *n* = 100, 86 (6), 80% female	No information about CI	Prospective 2 years F = 75, NF = 25	- Gait characteristics were not predictive of long-term falls
Buisseret, 2020 [64]	Prospective study Nursing homes Total *n* = 73, 83 (8) 62% female	CI included, 16% with dementia	Prospective 6 months F = 23, NF = 50	- When the Timed-Up and Go test results are coupled with indicators of gait variability measured during a six-minute walk test, accuracy of fall prediction improved from 68 to 76%
Ejupi, 2017 [60]	Cross-sectional Community-based Total *n* = 94, 80 (7), 68% female	CI as exclusion criterion according to MiniCog and MMSE	Retrospective 12 months F = 34, NF = 64	- F showed significantly lower maximum acceleration, velocity and power during sit-to-stand movements compared to NF (p < 0.05)
Gietzelt, 2014 [36]	Cohort-study Nursing homes Total *n* = 40, 76 (8), 50% female	CI included (MMSE 9.3 ± 8.0)	Prospective for 2, 4 and 8 months F = 13, NF = 27	- It is possible to classify gait episodes of F and NF for mid-term monitoring (4 months) during daily life using body-worn sensors (75.0% accuracy)
Greene, 2012 [55]	Prospective study Community-based Total *n* = 226, 72 (7), 73% female	CI as exclusion criterion	Prospective 2 years F = 83, NF = 143	- Sensor-derived features yielded a mean classification accuracy of 79.69% for 2-year prospective falls
Howcroft, 2016 [56]	Cross-sectional Community-based Total n = 100, 76 (7), 56% female	CI as exclusion criterion according to self-reports	Retrospective 6 months F = 24, NF = 76	- Best fall classification model using pressure-sensing insoles and head, pelvis and shank accelerometers (84.0% accuracy) - Best single-sensor model with parameters derived from a head sensor during single task (84.0% accuracy)
Howcroft, 2018 [57]	Prospective study Community-based Total *n* = 75, 75 (7), 59% female	CI as exclusion criterion according to self-reports	Prospective 6 months F = 28, NF = 47	- F had significantly lower dual-task head anterior-posterior Fast Fourier Transform first quartile, single-task left shank medial-lateral Fast Fourier Transform first quartile, and single-task right shank superior maximum acceleration (*p* < 0.05)
Hua, 2018 [41]	Cross-sectional Community-based Total *n* = 67, 76 (6), 100% female	No information about CI	Retrospective 1 year, Short Physical Performance Battery HFR = 19, LFR = 48	- Coefficient of variance, cross-correlation with anteroposterior accelerations, and mean acceleration were the top features for classification in HFR and LFR group
Ihlen, 2018 [44]	Prospective study Community-based Total *n* = 303, 76 (7), 50%	Including CI (MMSE≥19)	Prospective 6 months SF = 58, MF = 46, NF = 199	- Higher phase-dependent multiscale entropy of trunk acceleration at 60% of step cycle in F compared to NF (*p* < 0.05) - PGME has predictive ability of falls among SF
Ihlen, 2016 [49]	Cross-sectional Community-based Total *n* = 71, 78 (5), 65% female	Cognitively intact according to MMSE score (≥24)	Retrospective 12 months F = 32, NF = 39	- Refined composite multiscale entropy and refined multiscale permutation entropy of trunk velocity and trunk acceleration can distinguish between daily-life walking of F and NF (75.0–88.0% sensitivity, 85.0–90.0% specificity)
Iluz, 2016 [35]	Cross-sectional Community-based Older adults total *n* = 71, 78 (5), 65% females; Younger adults Total *n* = 30, 28 (4), 57% female	Cognitively intact according to MMSE score (≥24)	Retrospective 1 year F = 33, NF = 38	- Temporal and distribution-related features from sit-to-walk and sit-to-stand transitions during daily-life differed significantly between F and NF - Mean classification accuracy was at 88.0% and better than traditional laboratory assessment
Mancini, 2016 [45]	Cross-sectional, prospective Community-based Total *n* = 35. 85 (5), 66% female	Dementia as exclusion criterion according to Clinical Dementia Rating Scale and/or MMSE	Retrospective 12 months, prospective 6 months Retrospective analysis: SF = 12, RF = 7, NF = 16 Prospective analysis: F = 7, NF = 28	- Quality of turning (mean turn duration, mean peak speed of turning, mean number of steps to complete a turn) were significantly compromised in RF compared to NF (*p* < 0.05)
Marschollek, 2009 [61]	Cross-sectional Geriatric setting Total *n* = 110, 80 (−), 74% female	no information about CI	Retrospective n/a F = 26, NF = 84	- Pelvic sway while walking, step length and number of steps in TUG differed significantly between F and NF (p < 0.05) - Adding sensor-based gait parameters to geriatric assessment improves specificity in fall prediction from 97.6 to 100.0%
Marschollek, 2011 [62]	Prospective Geriatric setting Total *n* = 46, 81 (−), −	No information about CI	Prospective 1 year n/a	- Sensor-derived parameters can be used to assess individual fall-risk (58% sensitivity, 78% specificity) and identified more persons at fall risk than a conventional clinical assessment tool
Pozaic, 2016 [63]	Cross-sectional Community-based Total *n* = 136, 73 (6), 69% female	CI as exclusion criterion according to Screening of Somatoform Disorders (> 10)	Prospective 1 month Fn = 13, NF = 123	- Time and frequency domain-based features derived from a wrist-worn accelerometer on the dominant and non-dominant hand can significantly distinguish between F and NF (*p* < 0.05)
Qui, 2018 [50]	Cross-sectional Community-based Total *n* = 196, 72 (4), 100% female	No information about CI	Retrospective 5 years F = 82, NF = 114	- Sensor-based data distinguished accurately between F and NF (89.4% accuracy)
Rivolta, 2019 [19]	Cross-sectional Hospital setting Older adults total *n* = 79, 69 (17), − Younger adults total n = 11, 35 (−), −	No information about CI	Tinetti Assessment Tool HFR = 33, LFR = 46	- Sensor-based balance and gait features assessed during Tinetti Test differed significantly between individuals with HFR and LFR (p < 0.05) - Linear model and artificial neural network had a misclassification error of 0.21 and 0.11, respectively, in predicting Tinetti outcome
Sample, 2017 [58]	Cross-sectional Community-based Total *n* = 150, 76 (9), 59% female	No information about CI	Retrospective 12 months F = 59, NF = 91	- Sensor-based data collected during Timed-Up and Go resulted in a more sensitive model (48.1% sensitivity, 82.1% specificity) than including Timed-Up and Go time duration only (18.2% sensitivity, 93.1% specificity)
Senden, 2012 [51]	Cross-sectional Community-based Total *n* = 100, 77 (6), 56% female	CI as exclusion criterion	Tinetti Assessment Tool HFR = 19, LFR = 31, NFR = 50	- Walking speed, step length and root mean square had high discriminative power to classify the sample according to the Tinetti scale (76.0% sensitivity, 70.0% specificity).
van Schooten, 2015 [52]	Cross-sectional, prospective Community and residential care home Total *n* = 169, 75 (7), 54% female	CI included (MMSE≥18)	Retrospective 6 months; prospective 6 months Retrospective analysis: F = 60, NF = 109 Prospective analysis: F = 59, NF = 110	- Sensor-derived parameters of the amount of gait (number of strides), gait quality (complexity, intensity, smoothness) and their interactions can predict prospective falls (67.9% sensitivity, 66.3% specificity).
Wang, 2017 [46]	Prospective Community-based Total *n* = 81, 84 (4), 44% female	No information about CI	Prospective 12 months MF = 11, NF = 70	- Rate in stair descent was higher in MF than in NF (p < 0.05).
Weiss, 2011 [53]	Cross-sectional Community-based Total *n* = 41, 72 (7), 66% female	Cognitively intact according to MMSE score (≥24)	Retrospective 1 year F *n* = 23, NF *n* = 18	- Sensor-derived Timed-Up and Go duration was significantly higher in F compared to NF (p < 0.05) - Jerk Sit-to-Stand, SD and average step duration correctly classify 87.8% of F and NF (91.3% sensitivity, 83.3% specificity)
Weiss, 2013 [54]	Prospective Community-based Total *n* = 71, 78 (5), 65% female	Cognitively intact according to MMSE score (≥24)	Prospective 6 months F = 39, NF = 32	- Gait variability differed significantly between F and NF (p < 0.05);
Zakaria, 2015 [42]	Cross-sectional Hospital setting Total *n* = 38, 67 (7), 47% female	No information about CI	Timed-Up and Go HFR = 21, LFR = 17	- Sensor-derived parameters of Timed-Up and Go phases can classify into people at HFR and people at LFR.

SD: standard deviation, n: number, MMSE: Mini-Mental State Examination, HFR: high fall risk, LFR: low fall risk, CI: cognitive impairment, SF: single faller, MF: multiple faller, NF: non-faller, F: faller, NFR: no fall risk

### Detection of fall status

Classification into fallers and non-fallers or in older adults at high risk or low risk of falling was conducted using three different methods: retrospective assessment (e.g., fall history questionnaire), prospective assessment (e.g., fall diaries) or clinical assessment of fall risk (e.g., Tinetti Score, TUG, SPPB). Moreover, five of the studies combined two of these methods (Table 1). The majority of studies compared fallers and non-fallers. A faller was defined as a person having at least one fall over a certain period of time, usually the past or prospective 12 months. Eight studies compared older adults at high and low risk of falling [19, 40–42] or non-fallers and multiple fallers [43–46]. Multiple fallers were defined as participants that had fallen at minimum twice during the investigation period.

### Use of sensors to assess fall risk

To obtain the data, the included studies used between one and five inertial sensors. That were mainly located close to the centre of the body at the lower back [40, 42–54] or legs [43, 50, 55–58] of the participants. Less frequently used sensor locations were chest [19, 58–60], pelvis [41, 56, 57], waist [61, 62], foot [19, 45, 46], head [56, 57] and wrist [63]. The majority of the studies used sensor-derived data to distinguish between the different fall status groups or for fall classification during clinical testing (e. g. gait analysis under single or dual task conditions. Nine studies assessed walking and other related tasks during daily life, i.e. in homes of participants with a duration of three to eight days (Table 2).

**Table 2:** Use of body-worn sensors to assess fall risk

Assessment while sensor was used	Applied sensors (range of sampling rates in Hertz)	Body location	Assessed variables
gait analysis (between 7.62 and 400 m) [35, 40, 41, 43, 47, 48, 51, 56, 57, 62]	DynaPort, Trigno wireless systems, Locometrix, X16-1C, ActiGraph, GT3X+, Freescale, DAAF, ETB-Pegasus (30 Hz–296.3 Hz)	head, waist, lower back, pelvis	temporal and spatial gait variables, local dynamic stability variables, variables of gait symmetry, acceleration variables, angle variables
daily-life walking between three to eight days [35, 36, 44, 49, 52, 54, 59]	Senior Mobility Monitor, SHIMMER platform, DynaPort, Opal, BMA280 (50 Hz–128 Hz)	chest, lower back, wrist, upper legs, lower legs	temporal and spatial gait variables, variables of gait symmetry and gait variability, variables of gait complexity and gait smoothness, angle variables, acceleration variables
Timed-Up and Go Test [42, 53, 55, 58, 61, 62]	SHIMMER platform, Freescale, Opal, Mobi8 System, combined sensor (100 Hz–256 Hz)	chest, waist, lower back, upper legs, foot	temporal and spatial gait variables, angular velocity variables, energy variables, angle variables
Tinetti Test [19]	GENEActiv (50 Hz)	chest	temporal and spatial gait variables, balance variables
six-minutes walking test [64]	DYSKIMOT (100 Hz)	lower back	acceleration variables, variables of gait variability
**others**			
standardized protocol with walking and sit to stand transitions [60]	not specified (50 Hz)	around the neck	temporal gait variables, acceleration variables
specially developed test battery [50]	Xsens (100 Hz)	lower back, upper legs, lower legs	temporal and spatial gait variables, angle variables, angular velocity variables,
semi-unsupervised walking and stair ascent and descent [46]	Opal (128 Hz)	lower back, ankle	temporal gait variables, variables of gait variability, variables of movement vigour

All applied sensors contained an accelerometer, a gyroscope or a combination of both

### Classification models

Nineteen studies applied different types of machine learning models (e.g. receiver operating curves, Naïve Bayes, decision tree) and logistic regression analysis in order to correctly assign the study participants to the right category (e.g. faller and non-faller) using the sensor data. Besides sensor-derived variables, four studies also included height, body mass index, age [19, 54, 58], fall efficacy and information processing speed [50]. Prediction models achieved sensitivities between 48.1 and 91.3%, specificities between 66.3 and 100.0% and accuracies between 68.0 and 90.0% (Table 3). When comparing the analysed classification models of the different assessment conditions, the best model was found for daily-life data of three consecutive days with accuracy of 90.6%, sensitivity of 91.7% and specificity of 89.2% [35]. The models with sensor-derived data of laboratory assessment were on average not as precise, but accuracies, sensitivities and specificities were still acceptable (best in-lab data model [50]: accuracy = 89.4%, sensitivity = 92.7%, specificity = 84.9%).

**Table 3:** Fall risk classification models

Author	Model	Acc (%)	Sen (%)	Spe (%)
Bautmans et al. [40]	logistic regression analysis, ROC	77.0	78.0	78.0
Bizovska et al. [43]	logistic regression analysis, ROC	–	53.0	85.0
Buisseret et al. [64] ^a^	binary classification, ROC	85.7	50.0	73,9
Greene et al. [55]	ROC	79.7	73.1	82.6
Gietzelt et al. [36]	decision tree	75.0	78.2	71.2
Howcroft et al. [56]	support vector machine and neural networks	80.0–84.0	50.0–66.7	89.5
Hua et al. [41]	random forests	73.7	81.1	–
Ihlen et al. [44]	Partial Least Square Regression Analysis	76.0 (SF) 68.0 (MF)	71.0 (SF) 67.0 (MF)	80.0 (SF) 69.0 (MF)
Ihlen et al. [49]	Partial Least Square Discriminatory Analysis	–	59.0–88.0	77.0–92.0
Iluz et al. [35]	Ada Boost, Support Vector Machine, Bag, Naïve Bayes	87.1–90.6	83.8–89.2	87.2–94.4
Marschollek et al. [62]	logistic regression, classification model	70.0	58.0	78.0
Marschollek et al. [61] ^a^	classification trees	90.0	57.7	100.0
Qui et al. [50] ^a^	logistic regression, Naïve Bayes, decision tree, boosted tree, random forest, support vector machine	79.7–89.4	87.2–92.7	69.2–84.9
Rivolta et al. [19] ^a^	linear model, artificial neural network	–	71.0–86.0	81.0–90.0
Sample et al. [58] ^a^	stepwise logistic regression, max-rescaled R^2^ value	–	48.1	82.1
Senden et al. [51]	linear regression analysis, ROC	–	76.0	70.0
van Schooten et al. [52]	logistic regression analysis, ROC	–	67.9	66.3
Weiss et al. [54] ^a^	binary logistic regression analysis	71.6	62.1	78.9
Weiss et al. [53]	binary logistic regression analysis	87.8	91.3	83.3

^a^ These models also include data of clinical assessment (e. g. body mass index)

Acc: accuracy, Sen: sensitivity, Spe: specificity, ROC: receiver operating curve, SF: single faller, MF: multiple faller

### Results for individuals with CI

Since most of the included studies were conducted in a community setting, participants with severe CI are less likely to have participated. In addition to the only study that included individuals with severe dementia [36], five studies were conducted in a geriatric or hospital setting but provided no information concerning the cognitive status of their participants [19, 42, 48, 61, 62] and three more studies did not explicitly exclude participants with CI [44, 52, 64]. Overall, these nine studies may reveal information about the use and ability of sensors and sensor-derived data to distinguish between groups of fall status or to predict fall risk in a sample of older individuals with CI.

Six of the nine studies [36, 44, 48, 52, 62, 64] had a prospective design with between six- and 24-months follow-up. Three studies had a cross-sectional design [19, 42, 61] and collected sensor-derived data during clinical assessments. Sensors were placed at the lower back [44, 48, 52, 64], the shank [36], the waist [61] and the chest [19, 42] and sensor data were collected within seven [36, 44] or eight [52] days of daily-life, a 20-m gait analysis [48, 61], the TUG [42, 62], the Tinetti Test [19] or a walking test [64]. Only two studies gave information on how the sensor was applied to the participant’s body. In the study of Gietzelt et al. [36], sensors were applied by instructed nursing staff while in the study of van Schooten et al. [52] study participants had to attach the sensor by themselves.

For daily-life data of gait quality (e.g. gait velocity, step frequency) classification models of those studies including older adults with CI revealed accuracies between 68.0–76.0%, sensitivities of 67.0–78.2% and specificities of 66.3–80.0% [36, 44, 52] and therefore performed worse than the best model found for individuals without CI [35]. For sensor data collected during clinical assessments accuracies of 70.0–90.0%, sensitivities of 50.0–86.0% and specificities of 73.9–100.0% were achieved [19, 61, 62, 64].

### Quality assessment

All studies included in this systematic review used reasonable methodology (Table 4) measured with NOS. Most studies did not apply randomized stratified sampling. Furthermore, not all included studies controlled for age and sex differences or other important factors resulting in a lower evaluation of the category “comparability”. Overall, cross-sectional and prospective studies achieved a mean score of six stars out of ten and nine total stars.

**Table 4:** Evaluation of study quality according to Newcastle-Ottawa Scale

Cross-sectional studies	Selection (5 stars)	Comparability (2 stars)	Outcome (3 stars)	Total Score (10 stars)
Bautmans et al., 2011	★★★	★	★★★	7
Brodie et al., 2015	★★	★	★★★	6
Brodie et al., 2017	★★★	★	★★★	7
Ejupi et al., 2017	★★	★	★★	5
Howcroft et al., 2016	★★★	★	★★★	7
Hua et al., 2018	★★★★	★	★★★	8
Ihlen et al., 2016	★★	–	★★★	5
Iluz et al., 2016	★	★	★★★	5
Mancini et al., 2016*	★★★	★	★★★	7
Marschollek et al., 2009	★★★	★	★★★	7
Pozaic et al., 2016	★★★	★	★★★	7
Qui et al., 2018	★★★	★	★★★	7
Rivolta et al., 2019	★★★	★	★★★	7
Sample et al., 2017	★★★	★	★★★	7
Senden et al., 2012	★★★	★	★★★	7
van Schooten et al., 2015*	★★★	–	★★★	6
Weiss et al., 2011	★★	–	★★★	5
Zakaria et al., 2015	★★	–	★★★	5
**Prospective studies**	**Selection** **(4 stars)**	**Comparability** **(2 stars)**	**Outcome** **(3 stars)**	**Total score** **(9 stars)**
Bizovska et al., 2018	★★★	★	★★	6
Buckinx et al., 2015	★★	★	★★★	6
Buisseret et al., 2020	★★★	★	★★★	7
Gietzelt et al., 2014	★★	★	★★★	6
Greene et al., 2012	★★★	★	★★★	7
Howcroft et al., 2018	★★★	–	★★★	6
Ihlen et al., 2018	★★	★	★★	5
Marschollek et al., 2011	★★	★	★★★	6
Mancini et al., 2016 ^a^	★★	★	★★	5
van Schooten et al., 2015 ^a^	★★★	★	★★	6
Wang et al., 2017	★★	–	★★	4
Weiss et al., 2013	★★★	★	★★	6

^a^ Mancini et al. [45] and van Schooten et al. [52] had a mixed study design and were therefore considered for both types of study design

## Discussion

As a consequence of the aging process, falls are a major issue in geriatric populations and require special consideration in the design and conduct of effective physical exercise interventions. Therefore, a comprehensive understanding of motor performance is required to detect underlying fall risk factors more precisely. Assessment of motor performance in geriatric settings is usually based on scales, questionnaires and time-keeping, and wearable sensors may present a more objective and reliable approach. This systematic review provides an update of the existing body of literature concerning the assessment of fall risk factors in motor performance using wearable sensors with a special consideration of older adults with CI.

All studies included in this systematic review, except for one prospective study [48], found that sensor-derived data are successful in distinguishing between groups of faller status, or are useful in fall classification models. When classification ability of sensor data was compared to conventional clinical assessment, sensor-derived variables outperformed data of clinical assessment [56]. Wearable sensors may thus be considered a good alternative to conventional clinical assessment methods for fall risk assessment.

With regard to the setting of data collection, our review shows that data derived from both daily-life and clinical assessments was used to predict, classify or distinguish between groups of fall status. For in-lab sensor-based gait analysis, using the mean of at least two walks for more reliable data was recommended [40]. Furthermore, gait features may differ depending on walking distance [40] and longer walking distance in clinical assessment may better reflect everyday walking [57]. Nevertheless, sensor data of in-lab assessments might be biased because participants might be affected by the awareness of direct observation or cameras and therefore might not behave naturally (e. g. adjustment of gait) [35, 41, 45, 46]. Hence, daily-life data might better represent everyday functioning and fall-risk than data collected in an in-lab setting [35, 45, 65].

With regard to sensor wearing time, some studies comprised data collection from three up to eight consecutive days. A full week of recording sensor data may cover the range of motor performance of older adults better than a time span of only three days [54], however, drop-out rate may be higher and feasibility may worsen with increasing wearing time. In addition, it may be important to not only take into account sensor data from gait but also from different activities, like sit-to-stand transitions [52].

When assessing sensor data during daily-life, various environmental conditions cannot be controlled. Moreover, movement behaviour in daily-life does not follow a protocol, so the amount of sensor data might differ significantly between study participants [35]. In contrast, in a supervised setting (e. g. nursing homes or hospitals), all participants are assessed in the same facility and environmental conditions are standardized and comparable [48].

The placement of the sensors differed within the included studies. The most-often used sensor location was the lower back for which a high user acceptance was reported in previous studies [66]. However, Howcroft et al. [56] examined different sensor positions and concluded that sensors placed at the head or pelvis provided the best classification capability among single-sensor models. Only one study group used wrist-worn sensors for detection of sit-to-stand transitions, but the performance was comparable to studies using waist-worn devices [63]. An advantage of wrist-worn sensors might be the non-intrusiveness and the similarity to a wristwatch [67].

Several parameters of motor performance identified through sensor data may provide valuable information about motor deficits that are associated with fall risk, as well as indications for further fall prevention programs. Interestingly, sensor-derived parameters that were associated with fall risk were not associated with clinical fall risk assessments (e.g. TUG). This may indicate that not all fall-related movements can be detected by conventional clinical assessments [55], thereby highlighting the importance of body-worn sensors. To overcome the potential limitations of clinical assessments, a combination of daily-life sensor data and outcomes of clinical assessments to improve fall prediction was recommended [43, 44].

Although individuals with CI represent the group with the highest risk of falling in older adults, they are often excluded from studies examining sensor-based methods to assess fall risk. Therefore, the secondary aim of this systematic review was to provide practical recommendations for using sensors in fall risk assessment in individuals with CI. Since recording of data during daily-life provides slightly better results, this may be one approach to consider for individuals with CI. The daily-life recording in the included studies ranged from three to eight days and was considered feasible regardless of the cognitive status of included participants. Previous studies with individuals with CI and dementia also reported good feasibility of sensor-based data collection of up to three days [68–70]. Recording of daily-life data should thus be preferred to in-lab data collection as individuals with CI are more likely to be affected from test instructions or external distraction [71].However, individuals with CI may be less active during the day which may hamper collection of high-quality data [72].

Furthermore, it must be noted that both the location and the method of attachment of sensors appear to be of high importance when collecting sensor-based data on individuals with CI. The application of more than one sensor may provide more detailed information but is less practicable in this target group [19]. In addition, particularly in individuals with CI, researchers or instructed nursing staff need to be present to assume or supervise the placement and correct wearing position of the sensor [36, 61]. From a practical point of view, the location of the sensor should be carefully chosen, and clinicians and researchers may want to ensure that participants are not disturbed by the device [67, 73]. Moreover, researchers and/ or clinicians may need to consider technical aspects such as battery life span, data transmission or storage capacity when selecting an appropriate sensor for research or clinical practice [19].

Furthermore, some studies concluded, that additional information concerning other fall-risk related factors (e. g. age) might improve fall prognosis [36], and more studies are needed to examine the interplay between cognitive functioning and motor performance for fall risk assessment [45].

### Strength and limitations

To the best of our knowledge, this review was the first to particularly focus on, and to also provide practical implications for using body-worn sensors in fall risk assessment in individuals with CI. However, several limitations must be noted. For example, we included studies with different study designs, which may limit the comparability of findings between studies*.* Furthermore, regarding our secondary aim, we only identified one study particularly focusing on individuals with CI. Therefore, we also considered studies not explicitly excluding individuals with CI for our practical recommendations. Nevertheless, this limits our ability to make assumptions about the use and practicability of wearable sensors in persons with CI. More research is needed to address this important topic, particularly as individuals with CI exhibit more gait abnormalities such as asymmetry as compared to persons without CI. In addition, besides motor performance, cognitive abilities as well as other factors such as medication intake, mental health, or support from caregivers also play a significant role when assessing risk of falling [11]. However, this review solely focused on sensor-based characteristics of motor performance. Of note, wearable sensors are also widely used in fall detection which we did not address with our systematic review. Combining wearable sensors for both fall risk assessment as well as fall detection may thus be an effective prevention strategy in clinical settings.

## Conclusion

In conclusion, wearable sensors appear to be feasible tools to assess fall risk in older adults regardless of CI, in both an in-lab setting and during daily-life when measured for a period of up to eight days. Overall, sensor-derived data of daily-life were more useful in distinguishing between or predicting groups of faller status, indicating that the wide range of variables from daily-life data provides more valuable information about fall risk as compared to data collected in an in-lab setting. Similar results were observed when focusing on older adults with CI. Nonetheless, there exists a considerable lack of studies particularly examining sensor-based fall risk assessment in individuals with CI. Future research is needed to further specify which sensor-derived parameters of motor performance measured in daily life are most accurate and reliable predictors of fall risk. Furthermore, more research should focus on use of wearable sensors for fall risk assessment in older adults with CI to improve exercise programs for fall prevention.

## Supplementary Information

**Additional file 1.**

## LIST OF ABBREVIATIONS

CI: Cognitive impairment
NOS: Newcastle-Ottawa Scale
PRISMA: Preferred Reporting Item for Systematic review and Meta-Analysis
SPPB: Short Physical Performance Battery
TUG: Timed-Up and Go Test
