# Supplementary material for: Sensor-based fall risk assessment in older adults with or without cognitive impairment: a systematic review
Source: Eur Rev Aging Phys Act. 2021 Jul 9;18:15. doi: 10.1186/s11556-021-00266-w (PMC8272315; doi:10.1186/s11556-021-00266-w)
Supplement: Supplementary file 1 — Additional file 1. [file 11556_2021_266_MOESM1_ESM.docx]

# Additional file 1

# Search strategy

Details of the search strategy used are provided below.

### PubMed

((fall risk[Title/Abstract] OR fall risk factor*[Title/Abstract])) AND (sensor*[Title/Abstract] OR objectively measured[Title/Abstract] OR objective measurement[Title/Abstract] OR acceleromet*[Title/Abstract])

### Scopus

( TITLE-ABS-KEY ( fall AND risk OR fall AND risk AND factor* ) AND TITLE-ABS-KEY ( sensor* OR objectively AND measured OR objective AND measurement OR acceleromet* ) )

### Web of Science

(fall risk OR fall risk factor*) AND TITLE: (sensor* OR objectively measured OR objective measurement OR acceleromet*)
